# Growth monitoring and promotion uptake and its associated factors among mothers/ caregivers of children aged under two years in Wondo District, Ethiopia: Cross-sectional Study

**DOI:** 10.1371/journal.pone.0352894

**Published:** 2026-07-02

**Authors:** Tsehay Negash, Amelo Bolka, Assefa Philipos Kare, Fentaw Wassie Feleke, Tafese Bosha

**Affiliations:** 1 Department of Human Nutrition, School of Human Nutrition and Food Science Technology, Hawassa University, Hawassa, Ethiopia; 2 Department of Public Health, School of Public Health, Yirgalem Hospital Medical College, Yirgalem, Ethiopia; 3 Department of Public Health, Woldia University, Woldia, Ethiopia; Public Library of Science, UNITED KINGDOM OF GREAT BRITAIN AND NORTHERN IRELAND

## Abstract

**Introduction:**

Growth monitoring and promotion (GMP) can be used to address developmental halting before a child’s health deteriorates. However, there hasn’t been much research done on the GMP uptake in the Wondo district.

**Objective:**

The study aimed to evaluate GMP uptake and its associated factors among children aged under two years old in the Wondo District in 2024.

**Methods:**

A cross-sectional design was conducted using random sampling. The data were analysed using SPSS version 25. Descriptive statistics and logistic regression with a p-value of less than 0.05 were used.

**Results:**

GMP uptake was 14.2%. Household income above $32 monthly [AOR = 4.857, 95% CI: (2.009–11.744), read and write [AOR = 10.625, 95% CI: (2.667–42.334)], primary education [AOR = 9.067, 95% CI: (3.218–25.546)], secondary and above[AOR = 8.060, 95% CI: (2.952–22.007)], ANC follow up, [AOR = 2.871, 95% CI: (1.158–7.120)], health facility delivery [AOR = 4.037, 95% CI: (1.706–9.554)], PNC follow up [AOR = 4.110, 95% CI: (1.898–8.900)], maternal community conversation [AOR = 4.162, 95% CI: (1.974–8.775)], and family health cards utilization [AOR = 6.825, 95% CI: (2.935–15.867)] were significant variables.

**Conclusion:**

The GMP uptake was 14.2%. ANC follow-up, institutional delivery, PNC follow-up, family/child health card utilization, maternal community discussions, mother’s educational status, and household income were associated factors. Community conversations, family health cards, ANC, institutional delivery, and PNC follow-up can improve GMP services.

## Introduction

Child malnutrition is a prominent cause of morbidity and mortality among children under five years of age, contributing to a vicious cycle of poor health consequences [1]. It continues to be one of the most significant global public health issues, especially in low- and middle-income (LMIC) nations where access to resources and medical facilities is usually limited [2]. Stunting, wasting, and micronutrient deficiencies are some of the manifestations of malnutrition, which all have significant and frequently irreversible impacts on a child’s physical and mental development. These factors exacerbate health inequities by impeding a child’s capacity to grow and making them more vulnerable to infectious disease [3].

A healthy diet is essential for optimal physical growth, immune system function, and cognitive development throughout these critical years [4]. A child’s first 1,000 days of life, from conception to their second birthday, are generally regarded as a critical window for addressing malnutrition. The groundwork for long-term development, growth, and health is established during this time [5]. On the other hand, malnutrition during this crucial stage may result in permanent effects like decreased educational achievement, decreased adult economic productivity, and a higher chance of developing chronic illnesses in later life [6].

GMP is a key preventive health intervention designed to track child growth and detect early signs of malnutrition [7]. To monitor a child’s growth trajectory over time, it entails routinely measuring important growth indicators like height, weight, and mid-upper arm circumference (MUAC), which are then plotted on growth charts [8]. It enables medical professionals to spot a growth change from the usual curve as a critical early warning sign of undernutrition and take appropriate action before the problem gets worse. To promote the best possible growth and development of children, GMP uptake also gives healthcare professionals a platform to offer nutritional counseling to carers. This includes advice on proper feeding practices, dietary diversity, and hygiene [7].

Ethiopia has integrated GMP uptake into its national child health and nutrition policy to reduce under-five mortality and malnutrition [9]. The government has increased access to essential child health services, such as GMP uptake through the Health Extension Program (HEP), which is carried out in collaboration with international organizations [10]. This community-based approach ensures the widespread delivery of GMP uptake, particularly in rural and underserved areas, through a vast network of health posts, health centers, and frontline Health Extension Workers who are crucial in monitoring child development, providing nutritional counseling, and promoting preventive healthcare practices [11]. Better child health outcomes and early malnutrition identification have resulted from this increased accessibility and community involvement [12].

Despite GMP uptakes’ potential to enhance child health outcomes in Ethiopia, several complex issues prevent their optimal implementation and use [13]. Effective service delivery is hampered by inadequate healthcare infrastructure, undertrained healthcare professionals, and low carer understanding of the significance of routine uptakes of GMP [10]. Moreover, socio-cultural views, geographical distances to health facilities, financial constraints, and perceived poor service quality further limit participation [14]. These barriers collectively contribute to low GMP uptake rates, diminishing their impact on combating child malnutrition [15].

In Ethiopia, there are differences in the uptake of GMP across urban and rural areas, as well as between various socioeconomic categories [16]. Due to its remote settlements and high rates of child malnutrition, the Oromia Region confronts particular difficulties in establishing and maintaining the GMP uptake [17].

Although numerous studies have assessed the GMP uptake in Ethiopia, little research has been conducted specifically in the Oromia Region. Ethiopia’s undernutrition prevalence varies by region. The regions with the greatest rates of stunting are Afar (42%), Oromia (41%), Amhara (40%), and Southern Nations, Nationalities, and Peoples’ Region (SNNP) (40%). When it comes to the total number of stunted children, Oromia and Amhara have the largest percentage [17]. It is crucial to investigate trends in the GMP uptake and related issues. For this reason, this study sought to assess the uptake and its associated factors of GMP among children aged 0–23 months in Wondo District, Oromia Regional State, Ethiopia.

## Materials and methods

### Study area

The study was conducted in Wondo District, West Arsi Zone, Oromia Regional State, Ethiopia. The district is located approximately 271 km from Addis Ababa, the capital city of the country, and 19 km from Shashamene town. The district is situated at an altitude ranging from 1700 to 2300 meters above sea level. It is generally characterized by a warm climate with a mean annual maximum temperature of 19°C and a mean annual minimum temperature of 17°C. In the district, there are various water sources frequently used by the population for domestic purposes. The total population of the district is 100,834, with 49,913 (49.5%) males and 50,921 (50.5%) females. The district comprises eight rural kebeles, 14 health posts, and three health centers, namely Bussa, Shasha, and Bachil. Wondo District has 89 health workers and 25 health extension workers.

### Study design and period

A community-based cross-sectional quantitative study design was employed to assess the GMP uptake among children under two years of age in Wondo District, West Arsi Zone, Oromia regional state, Ethiopia. The study was conducted from May to July 2024.

### Population

The source of populations was all mothers and /or caregivers who had children aged 0–23 months and lived in the Wondo District, while the study populations were all mothers or caregivers who had children aged 0–23 months in chosen kebeles and who fulfilled the inclusion criteria during the study period. All child-mother or caregiver pairs who reside in Kebele and have children aged 0–23 months and who were at home during data collection were included. All mothers or caregivers who had children aged 0–23 months and had been residents of selected kebele in the district for at least 6 months before data collection began were included. Relative visitors and emigrants who moved into the district from their home location during data collection, however, were not included in the study.

### Sample size determination

The sample size was determined by considering the two specific objectives of the study.

### Sample size for first objective

Sample size for the first objective was calculated by using the single population proportion formula with the following assumptions:

Using Epi Info software version 7.2, the sample size for the first objective was calculated, accounting for the 95% confidence level, 5% margin of error, and the 53% of mother-child pairs that used GMP uptake [18] from a previous study conducted in southern Ethiopia [19].


$$\begin{array}{c} \text{n}=\frac{{\left(\text{Z}\frac{\alpha }{\text{2}}\right)}^{\text{2}}\text{ P}(\text{1}-\text{P})}{{\text{d}}^{\text{2}}}=\frac{\left(\text{1}.\text{9}\text{6}\times \text{1}.\text{9}\text{6})\text{0}.\text{5}\text{3}(\text{1}-\text{0}.\text{5}\text{3}\right)}{\left(\text{0}.\text{0}\text{5}\times \text{0}.\text{0}\text{5}\right)}=\text{3}\text{8}\text{3} \end{array}$$

The sample size determined for the first specific objective was 383.

### Sample size for the second objective

The sample size was also calculated for the second specific objective by using Epi Info version 7.2 software, considering 95% confidence level, a power of 80%, and a 1:1 ratio (Table 1).

**Table 1:** Sample size calculation for the second specific objective.

Factors	CL (%)	Power (%)	Ratio	% in unexposed	% in exposed	Odds ratio	Sample size	Reference
Maternal or caregiver place of delivery	95	80	1:1	10	33	3.01	224	[20]
GMP session distance	95	80	1:1	16	34.4	3.14	102	[21]
Maternal or caregiver education	95	80	1:1	52.4	35.5	1.99	306	[20]

Finally, the sample size was determined based on two objectives. For the first objective, a single population proportion formula (95% CI, 5% margin of error, and 53% GMP uptake) yielded 383 participants. For the second objective, sample size calculation for associated factors (95% CI, 80% power, 1:1 ratio) gave a maximum of 306 participants. Finally, the larger sample size (383) was selected and adjusted with a 10% non-response rate, resulting in a final sample size of 426.

### Sampling procedures

**Fig 1:**
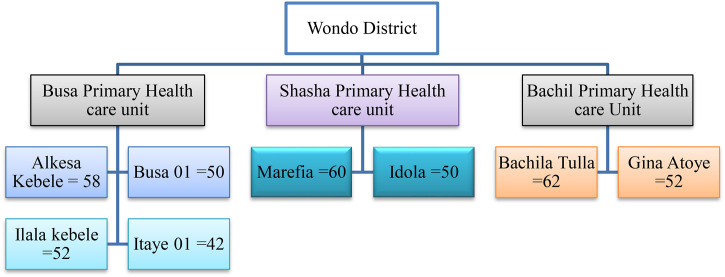
Schematic presentation of sampling procedure and participant selection.

The study participants were selected using a stratified random sampling process that was multistage. Out of the 14 available health posts in the Wondo district, eight were first selected by the lottery. Lists of all infants, young children (ages 0–23 months), and their mothers who lived in each of the selected health posts were supplied by the associated health posts. For each health post, sampling frames were created using these lists. A proportionate percentage of the 426-sample population was then allocated to the selected health posts. Finally, a simple random sample approach was used to select mothers or caregivers with infants or children under two years old from each health facility (Fig 1).

### Data collection instrument

Since these data result in generalizations, obtaining reliable and accurate data is a crucial step in the research process. Data were collected using pre-tested, structured, interviewer-administered questions from previous studies [19,22]. The pre-test was conducted on 22 samples (5% of the total sample). The tool has socio-demographic, household factors, and social factors addressing all the variables. The questionnaires were first prepared in English, then translated into the local language, called Afaan Oromo, and back translated into English to check consistency by language experts. The questionnaires were adopted from similar previously published studies. Eleven of the items were to assess socio-demographic characteristics, eight of the items were to assess maternal knowledge on GMP uptake, and twelve items were to assess health service-related factors.

### Data collection procedure

Three health officers who had previously collected data were employed as data collectors, and a public health supervisor who had previously supervised data collection was brought in to supervise the daily data collection procedure and ensure the quality of the data. The principal investigator also followed the usual data collection procedure. Data were collected after verbally explaining the study’s purpose, advantages, and objectives to each participant and getting their written consent. At the houses of the mothers and /or caregivers, interviews were conducted in Afaan Oromo.

### Operational definitions

#### GMP uptake.

Nutritional status was assessed using WHO 2006 Weight-for-Age (WFA) growth standards (Z-scores) recorded on the child’s growth monitoring WFA chart, and GMP uptake was verified using growth monitoring cards/family health cards given for each mothers having children under two years with documented WFA plotting and caregiver counseling, defined as at least one visit for 0 months, two visits for 1–3 months, five visits for 4–11 months, and four visits per year for 12–23 months [23].

#### Child food poverty.

Child food poverty is measured using the UNICEF and WHO dietary diversity score. To meet the minimum dietary diversity for healthy growth and development, children need to consume food from at least five out of the eight defined food groups (1. Breastmilk, 2. Grains, roots, tubers, and plantains, 3. Pulses, nuts, and seeds, 4. Dairy products, 5. Flesh foods (meat, poultry, and fish), 6. Eggs, 7. Vitamin A-rich fruits and vegetables, and 8. Other fruits and vegetables. If children were fed: 0–2 food groups per day, they are living in severe child food poverty, 3–4 food groups per day, they are living in moderate child food poverty, and 5 or more food groups per day, they are not living in child food poverty [17,24,25].

#### Minimum acceptable diet.

The proportion of children aged 6–23 months who were breastfed and who got at least two milk feedings, as well as the percentage of non-breastfed children aged 6–23 months who received at least two milk feedings and had at least the minimum dietary diversity (excluding milk feeds) and minimum meal frequency during the previous day [24,26].

#### Minimum dietary diversity.

This study defined children aged 6–23 months who received foods from five or more food groups from eight food groups [17,24,26].

#### Minimum meal frequency.

Children receiving complementary foods (solid, semi-solid, or soft foods) a minimum number of times or more among children 6–23 months. The recommended number of meals per day for 6–8 months, 9–11 months & 12–23 months is 2–3 times, 3–4 times, and 3–4 plus 1–2 snacks, respectively [17,24,26].

### Study variables

The utilization of GMP was the dependent variable, while sociodemographic (age, sex, ethnicity, religion, educational attainment, marital status), maternal (knowledge of GMP, attitude towards GMP), health facility-related (GMP availability, distance to health facility), and socioeconomic (family income level, mother’s occupation) factors were the independent variables.

### Data quality control

This is to identify and address any limitations or problems with the instruments and process used to collect the data. Considering the pretest results, the questionnaire was reviewed and refined for consistency, comprehensiveness, comprehensibility, and clarity. Before data collection began, supervisors and data collectors also received three days of training. The training covers topics such as study objectives, ethics, data-gathering techniques, interview procedures, questionnaire administration, and quality control. Supervisors received training on how to evaluate surveys for consistency and completeness.

The questions were also suitably categorized and classed during data entry. Coding and categorizing the questionnaire responses simplified data management, increased accuracy and consistency, streamlined analysis, enhanced interpretation, and preserved quality. Throughout the study, pretesting, training, coding, and ongoing supervision were all combined to maximize the quality of the data.

### Data entry and analysis

Epi-data version 4.6.2 was used to code and enter the data. After that, it was exported to SPSS version 25 for analysis and cleaning. Both independent and dependent variables were subjected to descriptive analysis. Text, tables, and graphs were used to calculate and display metrics such as mean, median, standard deviation, frequency, and percentage. To evaluate correlations between the independent and dependent variables, binary logistic regression was used. Candidates for the multivariable logistic regression model were variables with p < 0.25. The Hosmer-Lemeshow test was used to assess the final model’s fitness. Variance inflation factors were used to evaluate multicollinearity (VIF < 10). A variable was deemed statistically significant if its p-value was less than 0.05. The resulting model’s connections were quantified using adjusted odds ratios (AORs) with 95% confidence intervals.

### Ethical consideration

The study included human volunteers and took ethical considerations into account. An institutional review board (IRB) letter from the College of Medicine and Health Sciences was obtained on April 15, 2024, with reference number IRB/246/16, approving the performance of the study. Following receipt of an official document authorizing the project’s start, data collection began with the consent of the Wondo district administration. Every selected kebele official in the area where the study was carried out was given a letter of cooperation. Written informed consent was obtained from mothers or caregivers of children aged 0–23 months at the household level. The volunteers were given thorough information in their native language regarding the goals, methods, duration, possible risks, and benefits of the study. Respondents were guaranteed anonymity and informed that their data would only be used for the study. Confidentiality was ensured by utilizing distinct code numbers on the surveys rather than respondent names.

## Results

### Socio-demographic characteristics

A total of 416 mothers/caregivers of children aged 0–23 months were interviewed, yielding a 97.7% response rate. Half of the children were female (50.5%). Most mothers were under 29 years of age (63.2%), while 35.1% were aged 29–39 years. Regarding education, 34.9% had primary education, and 6.5% had college/University education. The majority of mothers (86.3%) were housewives. More than half of the children (67.5%) were aged 12–23 months, with a mean age of 13.9 ± 5.86 months and a median of 14 months. About 32.5% of households had a family size greater than five members, and 12.5% of fathers had attained secondary education (Table 2).

**Table 2:** Sociodemographic characteristics of mothers/caregivers in Wando district, Ethiopia, 2024.

Variables	Categories	Frequency	Percentage
Maternal or caregiver’s age in completed years	<29	263	63.2
	29-39	146	35.1
	>39	7	1.7
Index child in completed months	<3	25	6.0
	4-11	110	26.4
	12-23	281	67.5
Index child sex	Female	210	50.5
	Male	206	49.5
Maternal or caregivers’ marital status	Never married	1	0.2
	Living together	395	95.0
	Living separately	12	2.9
	Divorced	8	1.9
Maternal or caregivers’ educational status	No formal education	62	14.9
	Read and write	138	33.2
	Primary	145	34.9
	Secondary	44	10.6
	College and above/University	27	6.5
Maternal or caregivers’ occupation status	Housewife	359	86.3
	Self-employed	33	7.9
	Student	4	1.0
	Government employee	19	4.6
	Farmer	1	0.2
Paternal education	No formal education	45	10.8
	Read and write	116	27.9
	Primary	146	35.1
	Secondary	57	13.7
	College and above/University	52	12.5
Paternal occupation	Self-employed	115	27.6
	Student	5	1.2
	Government employee	46	11.1
	Farmer	250	60.1
Household family size	≤5	281	67.5
	>5	135	32.5
Household monthly income	≤ $32	244	58.7
	> $32	172	41.3

### Health service access and utilization by mothers or caregivers

The study showed that 81.5% of respondents attended ANC visits, with 65.6% having four or more visits. Regarding place of delivery, 64.9% of births occurred in health facilities, while 35.1% took place at home. PNC follow-up was reported by 44.5% of respondents. Overall, 72.8% had heard of GMP, mainly from health extension workers (53.6%) and other health workers (26.9%), while 3.1% cited media and 16.3% had not heard of GMP. GMP utilization was low at 14.2%, and 47.1% of respondents had a Family Health Card (Table 3).

**Table 3:** Mothers/caregivers’ healthcare services in Wando district, Ethiopia, 2024.

Variables	Frequency (n)	Percentage (%)
ANC visit		
Yes	339	81.5
Frequency of ANC follow-ups		
<4	143	34.4
≥4	273	65.6
Place of delivery		
Home	146	35.1
Health facility	270	64.9
PNC follow-up		
Yes	185	44.5
Heard about growth monitoring before?		
Yes	303	72.8
Source of information about GMP		
Health extension workers	223	53.6
Other health workers	112	26.9
Media	13	3.1
Not heard	68	16.3
GMP uptake		
Yes	59	14.2
Possession of Family Health Card		
Yes	196	47.1

### Growth monitoring and promotion uptake

**Fig 2:**
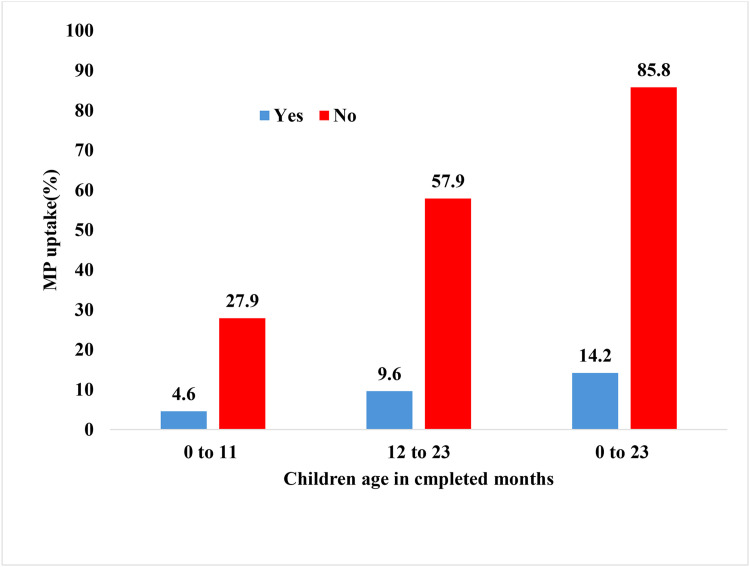
GMP uptake among children under two years by age in Wondo district, Ethiopia, 2024.

The GMP uptake was 14.2% with a 95% CI [11.0%−17.9%]. The uptake of GMP was lower among children aged 0–11 months(4.6%) compared to those aged 12–23 months (9.6%) (Fig 2).

### Factors associated with GMP uptakes

In the bivariable logistic regression analysis, maternal age, child age, place of residence, ANC follow-up, place of delivery, family size, maternal education, maternal workload, caregiver awareness of regular child weighing, decision-making for GMP uptake, distance from the health facility, husband support, child health service utilization, and community conversation (CC) were found to be significantly associated with GMP uptake and were entered into the multivariable logistic regression model. After adjustment, household income, ANC follow-up, place of delivery, PNC follow-up, maternal educational status, family/child health service utilization, and maternal participation in CC were found to be significantly associated with GMP uptake.

Mothers or caregivers with a household income above $32 per month had higher odds of GMP uptake compared to their counterparts (AOR = 4.857, 95% CI: 2.009–11.744). Mothers or caregivers who could read and write (AOR = 10.625, 95% CI: 2.667–42.334), had primary education (AOR = 9.067, 95% CI: 3.218–25.546), and had formal education (AOR = 8.060, 95% CI: 2.952–22.007) showed higher odds of GMP uptake compared to those who could not read and write.

Similarly, mothers or caregivers who had at least four ANC visits (AOR = 2.871, 95% CI: 1.158–7.120), institutional delivery (AOR = 4.037, 95% CI: 1.706–9.554), at least one PNC visit (AOR = 4.110, 95% CI: 1.898–8.900), participation in community conversations (AOR = 4.162, 95% CI: 1.974–8.775), and utilization of family/child health cards (AOR = 6.825, 95% CI: 2.935–15.867) were significantly associated with higher odds of GMP uptake (Table 4).

**Table 4:** GMP uptake-associated factors among children under two years in Wondo district, Ethiopia, 2024.

Variable	Category	GMP Uptake		95% CI		P- value
		Yes	No	COR	AOR	
Maternal age	Below 29	42	221	1	1	
	29 and above	17	136	1.520(0.832 - 2.777)	1.169(0.541 - 2.525)	0.691
Maternal or caregiver education	No formal education	5	57	1	1	
	Read and write	14	124	6.196 (2.199 - 17.455)	10.625(2.667- 42.334)*	0.001
	Primary	15	130	4.814(2.305 - 10.055)	9.067(3.218 - 25.546) ***	0.0001
	Secondary and above	25	46	4.710(2.286 - 9.706)	8.060(2.952 - 22.007) ***	0.0001
Household income	≤32 dollars	48	196	1	1	
	Above $ 32	11	161	3.584(1.802 - 7.129)	4.857(2.009 - 11.744) ***	0.0001
ANC follow-up	<4	8	135	1	1	
	4 and above	51	222	3.877(1.785 - 8.418)	2.871(1.158 - 7.120) *	0.023
Place of delivery	Home	11	135	1	1	
	Health facility	48	222	2.654(1.332 - 5.286)	4.037(1.706 - 9.554) *	0.002
Postnatal care	Yes	37	148	2.375(1.346 - 4.192)	4.110(1.898 - 8.900) ***	0.0001
	No	22	209	1	1	
Family size	Above 5	31	250	1	1	
	≤5	28	107	2.110(1.207-3.690)	2.135(0.975 - 4.674)	0.058
Maternal or caregiver CC participation	Yes	31	261	2.456(1.40-4.308)	4.162(1.974 - 8.775)***	0.0001
	No	28	96	1	1	
FHC utilization	Yes	50	146	8.029(3.829-16.835)	6.825(2.935 - 15.867) ***	0.0001
	No	9	211	1	1	

*P-Value<0.05, ***P-Value<0.0001, 1 reference.

## Discussion

The study revealed that the overall GMP uptake was 14.2% (95% CI: 11.0% – 17.9%), which is slightly consistent with findings from the Afar region of Ethiopia (15.9%) [27] and Mareka District in Southwest Ethiopia (16.9%) [15]. This similarity may be attributed to the use of comparable outcome measurement tools, similar age groups, sampling techniques, and study settings.

The current study finding is lower than Sude district Southeast Ethiopia [28], 47% in Decha district Southwest Ethiopia [29], North Gonder Northeast Ethiopia at facility level 50.4% [30], Metu town 25.2% [31], Sothern Ethiopia Muhir Akil district 32.9% [32], Ethiopia 23.21% [33], 25.71% [34], Digalo Tijo district of Oromia region 39.2% [21], Banja district 38.9% [20], Ghana reported 28.5% [35], Uganda 52.5% [36], Kenya 58.1% [37], Lawra district upperwest Ghana 53% [38], Ghana 60% [39], South Africa 67% [40], and 53% in 2004 and 79.7% in 2008 [41] reported in Rwanda. Variations in the magnitude of findings may be due to differences in study methodology, sociodemographic and cultural factors, GMP program implementation, access to services, health care quality, and study settings. The discrepancy with the study in Rwanda may be related to differences in sampling techniques. In addition, the presence of health extension workers (HEWs) providing regular home visits and the Sustainable Undernutrition Reduction in Ethiopia (SURE) program offering home-based nutrition counseling may have contributed to differences. Variations in study area, sample size, educational status, and distance to health facilities may also explain the observed discrepancies.

The finding of this study was higher than that of the study conducted in Butajira town (11%) [42]. This difference may be attributed to variations in study period, socioeconomic and demographic characteristics of participants, as well as differences in geographic coverage and study settings.

Monthly household income, ANC follow-up, place of delivery, PNC follow-up, educational status of mothers, family/child health card utilization, and maternal community conversation participation were found to be associated factors for the GMP uptakes.

Mothers or caregivers with a monthly household income exceeding $32 had higher odds of GMP uptake compared to their counterparts (AOR = 4.857). This finding is consistent with a study conducted in Mareka District, Southern Ethiopia [15], Banja [20], and Gonder Zuriya districts in northern Ethiopia [43]. This may be due to the fact that caregivers in remote areas may not perceive GMP services as essential or free of charge. In addition, mothers or caregivers in rural settings may give lower priority to GMP uptake. Limited financial resources may also lead families to prioritize daily income-generating activities over the long-term benefits of GMP services [44].

Mothers or caregivers with formal education were more likely to GMP uptake than mothers or caregivers without formal education in our study. This is again supported by the investigation conducted in Areka south Ethiopia [45], Butajira town [42], Afar region in northeast Ethiopia [27], Banja district in Northwest Ethiopia [20], Armachiho district in Northern Ethiopia [46], Ethiopia [34], Sude district Southeast Ethiopia [28], Ghana [47], and Uganda [36] and Nepal [48]. This is because more educated mothers are more likely to seek medical attention and possess greater health-related knowledge, which enhances the use of child health services, including GMP uptake [49,50]. This may be because mothers or caregivers with higher levels of education are more likely to be health-conscious and have been exposed to health knowledge, which enhances their behaviour while seeking medical attention and enables them to utilise GMP effectively.

The odds of ANC follow-up were three times higher among mothers or caregivers who had four or more ANC visits compared to their counterparts. This finding is consistent with a study conducted in Legambo District, South Wollo [51], and Ethiopia [29,33,34]. This may be because ANC visits provide counseling on maternal and child health, including nutrition and GMP uptake, which improves mothers’ or caregivers’ knowledge and supports informed decision-making regarding their children’s health. Mothers or caregivers who delivered in a health facility had higher odds of GMP uptake compared to those who delivered at home (AOR = 4.037). This aligns with research conducted in the Afar region [27] and Mareka and Decha districts in Southwest Ethiopia [15,29], Sude district in Southeast Ethiopia [28], Armachiho district in Northern Ethiopia [46], and Gonder Zuriya district in Northeast Ethiopia [43]. This may be because mothers or caregivers who deliver in health facilities are more likely to receive counseling on child nutrition, growth, and development compared to those who deliver at home. In addition, ANC visits provide opportunities for health education, including the benefits of GMP uptake. Mothers or caregivers who had at least one PNC follow-up were more likely to utilize GMP services compared to their counterparts (AOR = 4.110). This study is supported by research findings from Afar [27] and Benin [52]. This consistency may be explained by the fact that ANC and PNC services include counseling on child nutrition, feeding practices, and growth monitoring. In addition, mothers or caregivers receive dietary guidance during follow-up visits and become more aware of available health services, which may encourage them to participate in GMP uptake after delivery [53].

Mothers or caregivers who had used child/family health cards were 6.827 times more likely to GMP uptake than their counterparts. This outcome is in line with studies carried out in South Central Ethiopia’s Digelo Tijo district [22] and Northern Ethiopia’s Yilmana Densa district [54]. This is also consistent with the study revealed from Metu town, southwest Ethiopia [31] and Ethiopia [33,34]. Family/child health cards frequently include thorough, crucial information about maternity and child health and nutrition in an eye-catching, colored format and text-based manner. Because they have easy access to important health and nutrition information, mothers or caregivers may be inspired to adopt the GMP uptake as part of their commitment to their child’s well-being. Mothers or caregivers can be reminded to seek medical assistance for GMP uptake using a family health card. Healthcare practitioners can also utilise cards to educate and encourage mothers or caregivers the GMP uptake by emphasising the need to track their child’s development. According to this study, promoting the usage of FHC may be a useful tactic to boost the GMP uptake, which would enhance children’s growth and general wellbeing.

Lastly, compared to their counterparts, mothers or caregivers who were taking part in community discussions about the GMP uptake had 4.162 times higher odds. This is consistent with the central Ethiopian district of Muhir Aklil [55] and the southern Ethiopian district of Loka Abaya [56]. The rationale could be that taking part in community discussions affected attitudes, practices, and knowledge regarding the GMP uptake.

### Strengths and limitations of the study

This study could not establish causal relationships between variables, as exposures and outcomes were assessed simultaneously, making temporal ambiguity unavoidable. Therefore, it was not possible to determine whether exposure preceded the outcome. In addition, changes over time were not assessed due to the cross-sectional nature of the data. Moreover, since mothers or caregivers were asked to recall service utilization over the past two years, the study may be subject to recall bias.

## Conclusions

In this study, GMP uptake was 14.2%. ANC follow-up, institutional delivery, PNC follow-up, family/child health card use, participation in community conversations, maternal educational status, and household income were significantly associated with GMP uptake. To improve GMP uptake during the first two years of life, strengthening community- and facility-based interventions is essential, particularly through integrating community conversations with maternal and child health cards and reinforcing services during ANC, delivery, and PNC visits. Furthermore, future studies using mixed-methods and prospective designs are recommended to further assess GMP uptake within the universal health coverage framework and to explore programmatic implementation challenges in Ethiopia.

## Supporting information

S1 File GMP uptake manuscript source SPSS data. (SAV)

S2 File GMP uptake manuscript source STATA data. (DTA)

## Abbreviations

AOR: Adjusted Odds Ratio
CC: Community conversation
CI: Confidence Interval
GMP: Growth Monitoring and Promotion
HEWs: Health Extension Workes
NCHS: National Center for Health survey
NGO: non-governmental organization
SD: standard deviation
SNNP: Southern Nations, Nationalities, and Peoples’ Region
SPSS: Stastical Program for Social Science
SURE: Sustainable Undernutrition reduduction in Ethiopia
UNICEF: United Nation’s International Children’s Emergency Fund
WHO: World Health Organization
